# Serum cytokeratin 19 fragment in advanced lung cancer: could we eventually have a serum tumor marker?

**DOI:** 10.3332/ecancer.2014.394

**Published:** 2014-01-30

**Authors:** Ahmed El Bastawisy, Mahmoud El azzouny, Gamal Mohammed, Ahmad Awad allah, Eman Behiry

**Affiliations:** 1 Medical Oncology and Haematology, National Cancer Institute, Cairo University, Cairo 11796, Egypt; 2 Clinical Pathology, Benha University, Benha 13511, Egypt

**Keywords:** cytokeratin 19 fragment, lung cancer

## Abstract

**Introduction:**

Lung cancer is one of the most lethal malignancies; however, no serum marker has been routinely recommended until now.

**Methods:**

This is a prospective case control study including two groups of patients: Group I—patients with advanced lung cancer and Group II—patients with benign lung disease as control. Serum cytokeratin 19 (CK19) fragment levels were measured at baseline by real-time polymerase chain reaction before first-line chemotherapy. The CK19 cut-off taken was 15-cycle threshold. The primary end point was the comparison of high CK19 in cases and controls. The secondary end point was the correlation between high CK19 and progressive disease (PD), progression-free survival, and overall survival (OS) in advanced lung cancer patients.

**Results:**

A total of 30 patients with advanced lung cancer (16 non-small and 14 small cell lung cancer) and 15 patients with benign lung disease were included and followed up during the period from October 2008 to October 2011 with median follow-up of one and half years. High CK19 was found in 90% of lung cancer cases as compared with 7% in controls (*p* < 0.001). High CK19 was found in all cases showing PD (*p* = 0.04). One-year OS in high CK was 61% as compared with 33% in normal CK (*p* = 0.1).

**Conclusion:**

Serum CK19 fragment is a potential diagnostic and prognostic marker for advanced lung cancer.

## Introduction

Lung cancer is one of the most aggressive diseases, with disappointingly slow progress in outcomes despite the incorporation of a large number of new drugs [1]. Perhaps one of the most important open issues in lung cancer is the unavailability of validated serum tumour markers, which could assist in diagnosis and may spare the patient unnecessary interventional diagnostic procedures. Serum tumour markers can also serve a prognostic function when correlated to clinical outcome of the disease. Moreover, it could usher in a new era for more drug able targets, which may help to improve the outcome of advanced lung cancer. Several studies have investigated the usefulness of tumour markers in lung cancer mainly in staging, during post-therapeutic follow-up and for prognosis evaluation and even for early detection. Cytokeratin 19 (CK19) fragment was found to be more sensitive than other markers, such as carcinoembryonic antigen and squamous cell carcinoma (SCC)-related antigen [2]. High serum concentrations of CK19 fragment were mainly related to tumour burden and indicate a poor prognosis; however, no serum tumour marker has been recommended as a standard in lung cancer up until now [2, 3].

Again, assessment of diagnostic and prognostic value of CK19 in a Caucasian population in comparison with other studies of different ethnicities will help us to evaluate geographical differences between lung cancer patients.

## Aim of work

The primary end point was a comparison of high CK19 in cases and controls. The secondary end point was correlation between high CK19 and progressive disease (PD), progression-free survival (PFS), and overall survival (OS) in advanced lung cancer patients.

## Patients and methods

This was a prospective case control study including two groups of patients: Group I—patients with advanced lung cancer and Group II—patients with benign lung disease as control.

### Inclusion criteria

- The patient must have had a histologically confirmed diagnosis of lung cancer.
- Stages III-B and IV lung cancer.
- Controls have had benign lung disease diagnosed clinically.
- The patient was at least 18 years of age.
- The patient had a performance status of (ECOG Scale) ≤ 2.
- Life expectancy of at least six months.
- The patient had adequate bone marrow function (WBC count ≥ 3.0 × 109/L, ANC ≥ 1.5 × 109/L platelet count ≥ 100 × 109/L, haemoglobin level ≥ 9 g/L.
- The patient had adequate liver function; serum bilirubin < 1.5 X ULN, ALT and AST levels < three times normal values; ALT and AST levels < five times normal limits allowed in patients with known liver metastases.
- The patient had adequate kidney function; plasma creatinine level < 1.5 times normal value.

Patients should have had compliance, mental state, and geographic proximity that allowed adequate follow-up, and they had to provide written informed consent before any study-specific procedure. The study was conducted according to the Helsinki declaration and the guidelines for good clinical practice, and the local ethics committees approved the protocol.

### Exclusion criteria

Patients who were pregnant or breastfeeding.

Patients with active second malignancy at the time of the study.

Patients who were involved in another clinical trial at the time of the study.

### Treatment plan

Patients with non-small cell lung cancer (NSCLC) received the following chemotherapy regimen:

- Gemcitabine: 1000 mg/m2, IV on 250 cc NS over 30 min D1,8.
- Cisplatin: 80 mg/m2 D1, IV on 500 cc over 1 h with standard hydration.
- Patients with small cell lung cancer (SCLC) received the following regimen:
- Etoposide: 100 mg/m2, IV on 250 cc NS over 30 min D1–3.
- Cisplatin: 80 mg/m2 D1, IV on 500 cc over 1 h with standard hydration.

Every three weeks, up to six cycles in responding patients, patients with extensive stage SCLC showing any degree of clinical response received prophylactic cranial irradiation.

Evaluation was done every six weeks.

### Study assessment

Pretreatment assessment included complete medical history and physical examination.

Further assessment conducted within seven days before treatment included vital signs, performance status (ECOG), peripheral blood was tested for the presence of CK 19 mRNA positive cells by real-time polymerase chain reaction (PCR) and complete blood count with differential and full biochemical panel, including liver and renal function tests were performed and repeated before each treatment course.

Radiological evaluation was carried out, including a computerised tomography (CT) scan of the chest and U/S abdomen and pelvis. Additional radiological imaging such as bone scan was done if indicated (routinely in SCLC). Imaging was repeated every six weeks.

Evaluation was carried out according to response evaluation criteria in solid tumours as follows: a complete response was defined as complete disappearance of all known disease determined by two observations not less than four weeks apart. A partial response meant 30% or greater reduction of the product of the perpendicular diameters of all measurable lesions. Stable disease was defined as less than 30% reduction or less than 20% increase in tumour size. PD was an increase of more than 20% in the product of the perpendicular diameters of all measurable lesions or the appearance of new lesions.

### Post-treatment evaluation included

- Medical history and physical examination every three weeks.
- CBC and chemistry every three weeks.
- CT chest and upper abdomen every six weeks.
- Other investigations were done if indicated.

### Statistical methods

The SPSS package (version 17.0) was used for data analysis. Mean and standard deviation were reported to describe quantitative data. The chi-square and Fischer exact tests were used to evaluate the differences in the distribution of the variables. The Kaplan–Meier method was used to estimate the overall and PFS and the log rank test to evaluate differences in survival among groups. P-value of ≤ 0.05 was considered significant.

## CK19 Fragment Measurement Method

### Sampling

Five-millilitre venous blood sample was obtained by sterile venepuncture and delivered into ethylenediaminetetraacetic acid vacutainer tube used for the detection of CK19 positive cells by real-time PCR.

**Real-time PCR of CK19**

Three steps were done to reach quantitation:

(a) RNA purification;
(b) efficient synthesis of first strand cDNA from mRNA;
(c) quantitative real-time PCR.

1. RNA purification: GeneJET RNA Purification Ki (supplied by Fermentas—PureExtreme #K0731).
2. Efficient synthesis of first strand cDNA from mRNA: First Strand cDNA Synthesis (supplied by Fermentas—RevertAid H Minus #K1631):

(i) RNA quantity: 10 ng of total RNA to generate first strand cDNA as the initial step of a two-step RT-PCR protocol.
(ii) Primers: Synthesis of first strand cDNA was primed with oligo(dT)18 primer, Oligo(dT)18 primers cDNA synthesis from the poly(A) tail present at the 3′-end of eukaryotic mRNA.

**PCR amplification of first strand cDNA**

Two microlitres of the first strand cDNA synthesis reaction mixture were used as template for subsequent PCR in 50 µL total volume.

(a) Positive control first strand cDNA synthesis reaction. All components were mixed and briefly centrifuged after thawing, and kept on ice.
  1. Standard reagents shown were added into a sterile, nuclease-free tube on ice in the indicated order.
  2. The reaction tubes were mixed gently and centrifuged.
  3. For oligo(dT)18 was incubated for 60 min at 42 °C.
  4. The reaction was terminated by heating at 70 °C for 5 min.
  5. Brief centrifugation was performed then we proceeded with control PCR amplification.
(b) Control PCR amplification
  1. The cDNA generated was diluted with the control first strand cDNA reaction 1:1000 in water, nuclease free.
  2. All PCR reagents after thawing were gently vortexed and briefly centrifuged.
  3. A thin-walled PCR tube was placed on ice and standard reagents were added.
  4. PCR was performed in a thermal cycler with a heated lid.
  5. 10 µL of the RT-PCR product was loaded on 1% agarose gel. A distinct 496 bp PCR product was visible after ethidium bromide staining.

**Quantitative real-time PCR**

(a) The Maxima Probe/ROX qPCR Master Mix (2X) (supplied by Fermentas—RevertAid H Minus #K0231).
(b) Components of the kit were: 1-Maxima Probe/ROX qPCR Master Mix (2X): 2 × 1.25 mL 2-water, nuclease free: 2 × 1.25 mL.

## Results

A total of 30 patients with advanced lung cancer (16 NSCLC and 14 SCLC) and 15 patients with benign lung disease were included and followed up during the period from October 2008 to October 2011 with median follow-up of one and half years.

### Patients’ Characteristics

Table 1 summarises patients’ characteristics with regard to age, sex, smoking history, histology, type, and stage.

### Comparison between cases and control as regards CK19

**Figure 1:**
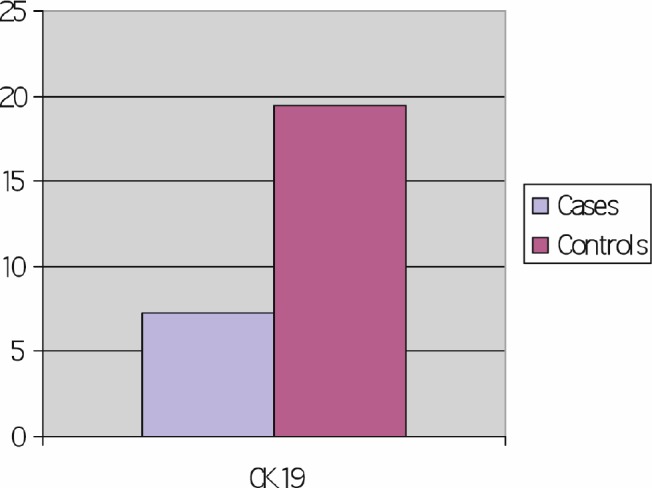
Comparison between cases and control as regards CK 19 (cycle threshold).

High CK19 was found in 90% of lung cancer cases as compared with 7% in controls. Cases of advanced lung cancer showed a statistically highly significant increase in CK 19 than benign lung disease. *P* < 0.001 (Figure 1).

There was significantly higher CK 19 in cases of NSCLC compared with cases SCLC, however, no difference was found between different histological types of NSCLC as high CK19 was found in 100% of each type (Table 2).

High CK19 was found in 17/19 (89.4%) of Stage III lung cancer as compared with 10/11(90.9%) denoting no significant correlation with stage.

### Clinical response

High CK19 was found in all cases showing PD and the results were statistically significant (*p* = 0.04), as shown in Table 3.

### Survival

**PFS**

The PFS could not be assessed statistically as all progressive cases had high values of CK 19.

**OS**

**Figure 2:**
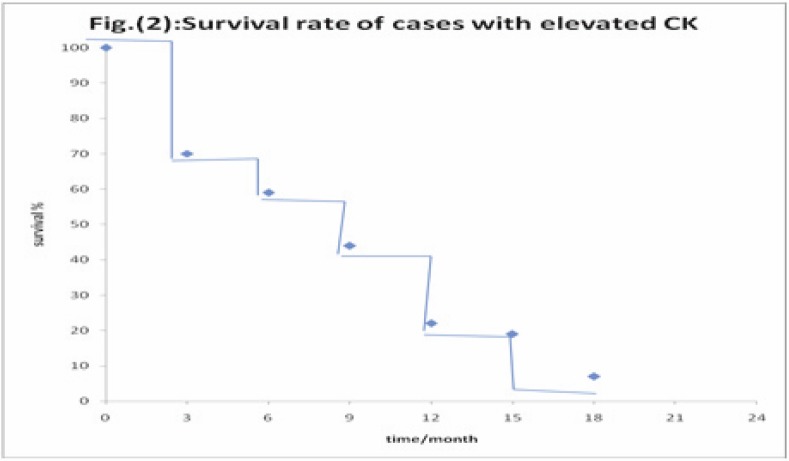
Correlation between high CK 19 and overall survival.

There was no statistically significant correlation between high CK19 and OS. The one-year OS in high CK19 was 61% versus 33% in normal CK19 (*p* = 0.1; Figure 2).

## Discussion

Lung cancer is a dismal disease with a poor outcome. Among methods to improve clinical outcome in lung cancer is to concentrate research not only on finding new drugs but also on methods of early diagnosis and monitoring of therapy. It is unacceptable in the era of personalised medicine that we do not have serum tumour makers for diagnosis or follow-up of lung cancer recommended in clinical practice. Thus, this study was designed as an attempt to discover a rapid and reliable method for the detection of advanced lung cancer rather than the ordinary invasive methods that had been used for diagnosis, again to define a prognostic value for this method. This study included two groups of patients:

Group I included patients with histologically confirmed lung cancer, and Group II included patients with non-malignant lung disease.

In our study, high CK19 was found in 90% of lung cancer cases as compared with 7% in controls. Cases of advanced lung cancer showed a statistically highly significant increase in CK19 than benign lung disease (*p* < 0.001). This result was in concordance with results reported by Weiskopf *et al* [4], Ebert *et al* [5], Paone *et al* [6], Krismann *et al* [7], Qu *et al* [16], and Wang *et al* [17].

Although our study showed significant increase in high CK19 in cases of NSCLC compared with SCLC as reported in a number of previous trials; however, there was no significant difference between different histological types of NSCLC in contrast to a number of previous trials, which showed higher incidence in SCC, this is because in our study all cases of NSCLC of different histology types showed high CK19. Again, our study showed no significant impact of lung cancer stage on correlation with high CK19.

In this study, high CK19 was found in all cases showing PD and the results were statistically significant (*p* = 0.04). The prognostic value of high CK19 was matched with the results reported by Zhao and Wang [9], Kasimir-Bauer *et al* [10], Chen *et al* [11], Sugio *et al* [12], Edelman *et al* [13], and Uchikov *et al* [14].

In this study, there was no statistically significant correlation between high CK19 and OS. One-year OS in high CK19 was 61% versus 33% in normal CK19 (*p* = 0.1). This is in contrary to the results reported by Bréchot *et al* [8] and Rosenblatt *et al* [15] who showed significant correlation with OS, this may be explained by the small sample size of the present study so more studies including larger numbers of patients and preferably separate study population of either SCLC or NSCLC are warranted.

Although the value of serum CK19 fragment was previously assessed in a number of trials, however, most of these trials included patients from the western or Chinese populations so the value of this study is that it shows the potential value of serum CK19 fragment in a Caucasian population like Egypt emphasising that geographic differences between lung cancer patients do not alter the diagnostic and prognostic value of CK19.

## Conclusion

In our study, it was concluded that high serum CK19 fragment is a potential tumour marker for advanced lung cancer carrying both diagnostic and prognostic value. This study aims at reattracting attention to focus research on the important topic of identifying serum tumour markers for lung cancer with the inclusion of a larger number of patients.

## Figures and Tables

**Table 1.** Patients’ characteristics.

Character	Cases Number(%)	Control Number(%)
Age	24–75 years (mean age = 59)	32–7 years (mean age = 45)
*Sex* Male Female	23(76.6) 7(23.4)	9(60) 6(40)
*Smoking* Yes No	21(70) 9(30)	8(53.3) 7(46.7)
*Histology* Adenocarcinoma Large cell carcinoma Squamous cell carcinoma Small cell carcinoma	7(23.3) 4(13.4) 5(16.6) 14(46.7)	–
*Type* COPD Pleural effusion IPS Empyema Pneumonia Haemoptysis	–	4(26.6) 6(40) 1(6.6) 1(6.6) 1(6.6) 2(13.3)
*Stage* III IV	19 11	–

COPD: Chronic obstructive pulmonary disease.

**Table 2.** Correlation between the type of the tumour and CK19

		*N*	Mean	Standard Deviation	SEM	Test of Significance	*P*
CK19 (ct)	SCLC	14	13.00	2.84	0.8	18.9	<0.001
	Large cell carcinoma	5	2.96	6.15	2.7		
	Adeno carcinoma	6	5.79	6.75	2.8		
	Squamous cell	5	−2.75	1.10	0.5		

CK19: Cytokeratin 19.

SCLC: Small cell lung cancer.

SEM: Standard error of the mean.

**Table 3.** Correlation between high, normal CK, and clinical response

*p*-Value	Normal CK	High CK	Clinical Response
0.047	Number of Cases	
	(0/14)0%	(14/14)100%	Progressive disease
	(2/9)23%	(7/9)77%	Stable disease
	(1/7)15%	(6/7)85%	Partial remission

CK: Cytokeratin.
